# *Chlamydia pneumoniae*: An Etiologic Agent for Late-Onset Dementia

**DOI:** 10.3389/fnagi.2018.00302

**Published:** 2018-10-09

**Authors:** Brian J. Balin, Christine J. Hammond, Christopher Scott Little, Susan T. Hingley, Zein Al-Atrache, Denah M. Appelt, Judith A. Whittum-Hudson, Alan P. Hudson

**Affiliations:** ^1^Department of Bio-Medical Sciences, Center for Chronic Disorders of Aging, Philadelphia College of Osteopathic Medicine, Philadelphia, PA, United States; ^2^Department of Biochemistry, Immunology and Microbiology, Wayne State University School of Medicine, Detroit, MI, United States

**Keywords:** late-onset dementia, Alzheimer’s disease, amyloid, *APOE*, *Chlamydia pneumoniae*, etiology, infection, neuroinflammation

## Abstract

The disease known as late-onset Alzheimer’s disease is a neurodegenerative condition recognized as the single most commonform of senile dementia. The condition is sporadic and has been attributed to neuronal damage and loss, both of which have been linked to the accumulation of protein deposits in the brain. Significant progress has been made over the past two decades regarding our overall understanding of the apparently pathogenic entities that arise in the affected brain, both for early-onset disease, which constitutes approximately 5% of all cases, as well as late-onset disease, which constitutes the remainder of cases. Observable neuropathology includes: neurofibrillary tangles, neuropil threads, neuritic senile plaques and often deposits of amyloid around the cerebrovasculature. Although many studies have provided a relatively detailed knowledge of these putatively pathogenic entities, understanding of the events that initiate and support the biological processes generating them and the subsequent observable neuropathology and neurodegeneration remain limited. This is especially true in the case of late-onset disease. Although early-onset Alzheimer’s disease has been shown conclusively to have genetic roots, the detailed etiologic initiation of late-onset disease without such genetic origins has remained elusive. Over the last 15 years, current and ongoing work has implicated infection in the etiology and pathogenesis of late-onset dementia. Infectious agents reported to be associated with disease initiation are various, including several viruses and pathogenic bacterial species. We have reported extensively regarding an association between late-onset disease and infection with the intracellular bacterial pathogen *Chlamydia* *pneumoniae*. In this article, we review previously published data and recent results that support involvement of this unusual respiratory pathogen in disease induction and development. We further suggest several areas for future research that should elucidate details relating to those processes, and we argue for a change in the designation of the disease based on increased understanding of its clinical attributes.

## Introduction

A longstanding idea in the medical literature is that a wide variety of chronic diseases could be caused or exacerbated by a microbial infection. For example, in the early 20th century rheumatoid arthritis was considered to be an infectious disease, an explanation that was more or less abandoned by mid-century but which has re-emerged (Lansbury, 1950; Ford, 1963; Albert, 2000; Carty et al., 2004). Multiple sclerosis long has been attributed at least in part to involvement of an infectious component, although the identity of the specific agent(s) remain(s) to be firmly established (Swanborg et al., 2003). Similar arguments have been made in regard to other chronic diseases of an idiopathic nature, but in most cases, an infectious etiology and/or involvement of microbes in disease exacerbation have been difficult to establish. The pattern is paralleled in cancer etiology, in which infectious causation of some cancers has been demonstrated (for review see de Martel et al., 2012); however, most cancers are not thought to have an infectious etiology.

Instead of viral, bacterial, or mycological involvement in disease causation, alternative mechanisms that might explain chronic disease genesis have been pursued. Among the most prominent explanations has been the potential genetic basis of chronic clinical entities, including what is termed late-onset Alzheimer’s disease. Most of these studies have indicated that development of disease is not easily attributable to one or even a few mutations or gene polymorphisms. Rather, such studies have indicated that disease genesis is multifactorial, resulting from as yet unknown environmental and host genetic factors (Harney and Wordsworth, 2002; O’Connor et al., 2006).

With regard to Alzheimer’s disease, the single idea that has predominated for almost three decades in studies of the neuropathology underlying this clinical entity is the “amyloid cascade” hypothesis. The hypothesis proposes that the deposition of the amyloid-β (Aβ) peptide is the critical event underlying neuronal degeneration, and thus cognitive dysfunction (Schellenberg, 1995). This idea is appropriately applicable to the early form of dementia, familial Alzheimer’s disease, since it is well established that this type of dementia is caused by genetic mutations resulting in an increase in amyloid formation and deposition (Tanzi, 2012). However, many studies have determined that causation of late-onset disease is not attributable to a small number of identical, similar, or other genetic defects leading to Aβ deposition, thereby undermining the contention that Aβ is the universal etiologic factor eliciting the dementia. Importantly, in both familial Alzheimer’s disease and late-onset disease, the neuropathology is essentially identical; this clearly indicates that factors other than the genetic lesions which underlie familial disease must exist to explain the neuropathology of late-onset disease.

Late-onset dementia typically is observed in older age; indeed, age is the primary risk factor for its development. Other factors also may increase risk of late-onset disease. These include: atherosclerosis (de la Torre, 2006), Type 2 diabetes (Revill et al., 2006), neurotrauma (Szczygielski et al., 2005) and infection (Miklossy, 1993; Itzhaki et al., 1997; Balin et al., 1998). Intriguingly, many of the same risk factors could promote systemic inflammation that may also influence disease pathogenesis. Furthermore, chronic, persistent, or latent infections in the brain, all of which may be an outcome following infection with a variety of organisms (see Itzhaki et al., 2004), could reactivate to initiate and/or exacerbate late-onset disease. These possibilities implicate both systemic and neuroinflammation in late-onset dementia. Thus, a likely scenario for development of late-onset dementia centers on poorly understood interactions between genetic risk, as exemplified in part by possession of the Apolipoprotein E (*APOE*ε)4 allele, and environmental factor(s), including infection. For these and other reasons, we have argued that late-onset disease is distinguishable from the genetically-based early-onset disease. On that basis we suggested the designation “late-onset dementia of the Alzheimer’s type” for the late-onset clinical entity, since it is more consistent with our current knowledge of the clinical and neuropathological underpinnings of the disease (Balin et al., 2017); in this article, we employ that designation, or simply late-onset dementia, throughout.

Many attempts have been made to identify infectious agents responsible for late-onset dementia of the Alzheimer’s type (see e.g., Emery et al., 2017). However, none of the agents studied has been unequivocally accepted to be either etiologic, or indeed exacerbating, for dementia-related neuropathology. These include many viral pathogens such as measles virus, lentiviruses, adenovirus and others (Pogo et al., 1987; Friedland et al., 1990). Importantly, studies of herpes simplex virus type 1 (HSV-1) infection in late-onset disease identified this virus as a risk factor for people expressing *APOE*ε4 (Itzhaki et al., 1997, 2001). Further, bacterial pathogens, including *Chlamydia trachomatis, Coxiella burnettii* and others have been investigated, but no clear relationship with Alzheimer’s pathogenesis has been demonstrated to date (Renvoize et al., 1987). In contrast, we discovered an association between the genesis of late-onset disease and infection with the intracellular respiratory bacterial pathogen *Chlamydia* *pneumoniae*. (Balin et al., 1998; Gérard et al., 2006). We summarize that work in this review, along with recently published studies from our group and those of others’ that implicate involvement of this unusual and ubiquitous pathogen in the genesis of senile cognitive dysfunction reflective of late-onset disease.

## Chlamydia Pneumoniae

*C. pneumoniae*, an obligate intracellular bacterium, is a pathogen of the respiratory tract, infecting mucosal surfaces, specifically the lung/pulmonary and nasal mucosa (Grayston et al., 1990; Campbell and Kuo, 2002; Hahn et al., 2002). It is ubiquitous in all societal cultures and geographic regions studied to date (Leinonen, 1993). Many reports have demonstrated that the organism is responsible for a significant proportion of community-acquired pneumonia, and it has been associated with numerous other pulmonary diseases (Grayston et al., 1990; Clementsen et al., 2002). Interestingly, *C. pneumoniae* infections also have been linked with an array of non-respiratory diseases, including atherosclerosis, inflammatory arthritis, multiple sclerosis and others (Sriram et al., 1998; Schumacher et al., 1999; Wagner et al., 2000; Belland et al., 2004). While some such associations are certainly controversial, credence has been gained for the role of this organism in atherogenesis during the last 20 years (Grayston, 2000; Rosenfeld et al., 2000; Belland et al., 2004).

Similar to other chlamydial species, *C. pneumoniae* displays a biphasic developmental cycle. The first phase involves the infectious extracellular form of the organism, the elementary body (EB). EB typically infect epithelial cells, but many cell types can be infected, including cells in the nervous system such as astroglia, microglia and neurons (Hatch, 1999; Dreses-Werringloer et al., 2006; Gérard et al., 2006). Following endocytosis into a cytoplasmic inclusion, EB reorganize into a reticulate body (RB), which is the metabolically active form of the organism. RB undergo multiple cycles of cell division followed by a “dedevelopmental” process yielding the infectious EB form again. These are released *via* eukaryotic cell lysis or exocytosis, which spreads the infection (Hatch, 1999).

Systemic dissemination of the organism from the respiratory tract has been well-documented (Gieffers et al., 2004), and several studies have indicated that this occurs *via* monocytes (Moazed et al., 1998). Importantly, *C. pneumoniae*, like other chlamydial species, undergoes long-term infection in a biological state termed “persistence” (Hogan et al., 2004). In persistence, the organism, as with other chlamydial species, is viable and metabolically active but does not complete its normal developmental cycle. Further, persistent chlamydial infections have been shown to be refractory to antibiotic treatment (e.g., Deniset and Pierce, 2010; Phillips-Campbell et al., 2014).

## Initial Studies of *C. Pneumoniae* and Late-Onset Disease

Our initial studies of possible involvement of *C. pneumoniae* in late-onset disease identified DNA of the organism in 90% of postmortem brain samples from patients diagnosed with late-onset dementia of the Alzheimer’s type; this study employed highly specific polymerase chain reaction (PCR) assays (Balin et al., 1998; Schumacher et al., 1999). Only 5% of control non-demented brain samples contained *C. pneumoniae* DNA. Positive DNA results were obtained from brain tissues from areas that normally display characteristic neuropathology (e.g., hippocampus) and from those less often demonstrating characteristic pathology (e.g., cerebellum). In nearly 90% of affected brain samples, positive PCR signals were obtained from at least one area showing neuropathology, and from the cerebellum in four cases. In these four cases, neuropathology existed within the cerebella as well as other areas. In contrast, the two relevant affected brain samples that failed to give a positive PCR signal for *C. pneumoniae* DNA exhibited only mild pathology (Balin et al., 1998). We further analyzed frozen brain samples for intact bacterial RNA using reverse transcriptase-PCR (RT-PCR). mRNA species encoding the KDO transferase and a ~376 kDa protein specific to *C. pneumoniae* were successfully targeted.

In these initial studies, immunohistochemistry and electron microscopy also were used to analyze samples from PCR-positive brains. Samples from individuals with clear late-onset disease contained *C. pneumoniae* antigens, specifically in cortical regions such as the temporal, parietal and pre-frontal cortices as well as the hippocampus. In these regions, organism immuno-positivity was observed in various cell types including perivascular macrophages, microglia and astroglia. Ultrastructural analysis of the positive brain samples revealed chlamydial inclusions containing both EB and RB. Immuno-electron microscopy using a gold-conjugated monoclonal antibody specific to an outer membrane protein demonstrated gold particles labeling the organism (Balin et al., 1998; Arking et al., 1999). Immuno-electron microscopy was negative in comparable PCR-negative control brain sections.

PCR and RT-PCR positive sample homogenates were prepared and incubated with human THP-1 monocytes in culture. Viable bacteria were successfully recovered from two brains positive for the organism. Culture of homogenates from two control brains prepared in a similar fashion generated negative results (Balin et al., 1998). Thus, DNA and antigens of *C. pneumoniae* were found in areas of neuropathology in brain samples from late-onset disease individuals, and samples from frozen brain tissues from these subjects yielded viable organisms. Genetic analyses revealed that 11 of the 17 PCR-positive samples had at least one allele for the *APOE*ε4 isoform (64%), consistent with that allele type being a risk factor for development of late-onset dementia (Roses, 1996). Importantly, in a separate study, it was shown that in patients with reactive arthritis who had DNA from *C. pneumoniae* in synovial tissues, 2/3 had one or more copies of the *APOE*ε4 allele (Gérard et al., 1999). Together, these and other observations discussed below clearly implicate a relationship between the *APOE*ε4 allelic genotype and infection by *C. pneumoniae*; they suggest that both factors confer an increased risk for chronic disease genesis (Balin et al., 1998; Gérard et al., 1999).

Not surprisingly, these initial studies and their implications suggesting that an infection with a common bacterium is involved in the origin of late-onset dementia instigated several other groups to attempt confirmation of the organism in brain tissues from other affected and control patient cases. Those studies yielded mixed results, with some reports providing positive identification (e.g., Mahony et al., 2000; Ossewaarde et al., 2000; Di Pietro et al., 2013a), and others failing to find DNA or antigens from the organism in relevant samples (e.g., Nochlin et al., 1999; Gieffers et al., 2000; Ring and Lyons, 2000; Taylor et al., 2002). Importantly and as reviewed by us previously in detail, numerous and various different techniques were used in the confirmation studies, with none utilizing methodology identical to our own. For example, one study failed to find *C. pneumoniae* in paraffin-embedded brain tissues from confirmed late-onset dementia patients *via* PCR or immunohistochemical (IHC) analyses (Nochlin et al., 1999); similarly, the organism was not found in several relevant paraffin-embedded brain samples using either PCR or IHC (Gieffers et al., 2000). The recovery of reasonable quality template for PCR from paraffin-embedded tissue can be unreliable, which might explain some negative results. We used exclusively frozen brain-tissue samples were in our studies. One report indicated success in identifying *C. pneumoniae* by *in situ* hybridization in many relevant brain samples (Ossewaarde et al., 2000); controls were negative, including those from individuals with other neurological diseases. In our studies, in negative IHC studies, and in the positive study, the same mAb targeting the *C. pneumoniae* MOMP or lipopolysaccharide (LPS) were employed, suggesting differences in technique in obtaining positive results. Another report explains the positive and negative PCR data from some studies (Mahony et al., 2000). This study employed replicate PCR assays and probit regression analyses to show that DNA from most frozen brain samples analyzed from relevant patients was PCR-positive for *C. pneumoniae* if enough replicates were performed; multiple assayed controls were always PCR-negative. Clearly, discrepancies in analytical methods used among the different laboratories severely constrain the results obtained (Campbell and Kuo, 2004).

### Confirmatory Studies

In subsequent research to confirm and extend our initial studies, we analyzed new tissue samples from late-onset dementia and control brains (Gérard et al., 2006). PCR analyses targeting two *C. pneumoniae* genes showed that samples from 20/25 late-onset brains, but only 3/27 control brains, were PCR-positive (Gérard et al., 2006). Viable organisms were cultured from the former brain samples, and metabolic activity of the cultured bacteria was demonstrated *via* identification of several intact chlamydial transcripts. Immunohistochemistry of relevant brain samples identified that astroglia, microglia and ~1/5 of neurons were infected with *C. pneumoniae*. As in our initial study, infected cells were located in the brain in proximity to both senile plaques and nerve cells containing neurofibrillary tangles (Balin et al., 1998). Together, these observations suggested that *C. pneumoniae* infection had a direct effect on neuronal cell injury/death. Further, the potential for the organism to act as initiator of granulovacuolar degeneration, another previously acknowledged pathology in late-onset disease, was suggested (Funk et al., 2011).

Continued analyses demonstrated that immunolabeling was positive for *C. pneumoniae* in the entorhinal and frontal cortices, and in the hippocampal formation of all relevant affected brains (Hammond et al., 2010). These areas exhibited both amyloid deposition and *Chlamydia* immunoreactivity in apposition to one another when stained with Thioflavin S and labeled with anti-*C. pneumoniae* antibodies on the same sections, thus revealing fibrillary amyloid and chlamydial immunoreactivity, respectively. Two extracellular patterns of chlamydial immunoreactivity were observed: a punctate pattern and a pattern with amorphous foci. These may represent extrusion of whole organism (punctate) or secreted chlamydial products, e.g., LPS (amorphous foci), into the tissues (Stuart et al., 1994; Hybiske and Stephens, 2007). Amyloid is known to have anti-microbial properties, perhaps allowing it to act as an anionic defensin (Bishop and Robinson, 2004; Kammerman et al., 2006; Soscia et al., 2010; Kumar et al., 2016). As reflected by the deposition of amyloid in the same region as *C. pneumoniae*, tropism of this organism to the central nervous system (CNS) may be a precursor or trigger for development of damage (Hammond et al., 2010).

The entorhinal cortex and the hippocampal formation, both olfactory structures, are known to be regions demonstrating the earliest damage in the late-onset brain (Mann et al., 1988; Christen-Zaech et al., 2003). Relevant to this, *C. pneumoniae* has been identified in both human and animal olfactory bulbs in experimental systems (Balin et al., 1998; Little et al., 2004, 2005, 2014). In animals, the organism appeared to spread centrifugally from the olfactory bulbs into the brain proper (Itzhaki et al., 2004; Little et al., 2004, 2005; Little et al., 2014). Thus, these and other observations outlined below support the idea that infection by this pathogen is an early event in the triggering of neuropathogenesis and not a consequence of prior damage providing access of infection to the CNS.

As indicated, *C. pneumoniae* is a respiratory pathogen, and both olfactory and lung routes for infection of the CNS are supported by DNA sequencing studies in which the organism isolated from a late-onset brain sample was shown to be more closely related to respiratory than to atherosclerotic strains (Roulis et al., 2015). We prepared two cultures of *C. pneumoniae* from infected brain tissues and evaluated genetic and cell biological features for both. As with most respiratory isolates, both of these isolates were genetically diverse (i.e., not clonal), and single nucleotide polymorphism (SNP) analysis indicated a number of differences even from standard respiratory isolates and strains (Dreses-Werringloer et al., 2009). However, we could not identify any genetic attributes that would indicate neuro-tropism. The recent full genome sequencing of one of the brain isolates in another laboratory confirmed this initial observation (Roulis et al., 2015). Cell biological studies of both isolates demonstrated that they showed standard inclusion morphology and typical chlamydial morphology upon culture in human epithelial cells (HEp-2), astrocytes (U-87 MG) and microglial cells (CHME-5), as in a prior publication (Dreses-Werringloer et al., 2006).

## *C. Pneumoniae* and *Apoe*ε4

apoE was initially identified as a protein component of very low density lipoprotein (VLDL) complexes (Shore and Shore, 1973). The gene encoding APOE is found on human chromosome 19 and is extensively expressed in many tissues. This locus has five allele types: ε2, ε3 and ε4 alleles are the most common in most groups studied, and some data indicate that the ε4 allele type is ancestral. Many studies have demonstrated that apoE is involved in cholesterol homeostasis and metabolism through the direction of the metabolic handling of triglycerides and cholesterol (Mahley and Rall, 2000). The ε4 allele product, apoEε4, is associated with increased risk for several diseases, including Alzheimer’s disease, atherosclerosis and others (e.g., Swanborg et al., 2003; Yu et al., 2014).

Importantly, *in situ* hybridization analyses targeting apoE showed that brain regions of ε4-bearing individuals with late-onset dementia contained a higher number of *C. pneumoniae*-infected cells as compared to congruent brain regions from individuals lacking the allele (Gérard et al., 2005). Real time PCR analyses of brain tissues targeting DNA sequences from the organism showed that although the bacterial load in samples lacking the ε4 allele varied, the samples from ε4-bearing individuals had higher loads than did comparable samples from those lacking the allele (Gérard et al., 2005). These observations are consistent with a role for the ε4 gene product in enhancement of dissemination of the organism from the pulmonary system, although we are unaware of studies specifically targeting this issue; this seems an area of interest for future research. Unlike apoE2 and apoE3, apoE4 appears to enhance attachment of *C. pneumoniae* EB to astroglia and microglia by several-fold over levels observed in the absence of that allelic product (Gérard et al., 2008). When adherent to the chlamydial EB, apoE4 retains its ability to attach to its receptor, the LDL receptor and other members of this receptor family, on the surface of host eukaryotic cells. Thus, while much remains to be elucidated regarding the relationship between apoE4 and enhanced *C. pneumoniae* uptake into individuals with this phenotype, the link between infection, apoE4, and diseases associated with both, including late-onset dementia of the Alzheimer’s type, is strengthened by our observations.

## Neuroinflammation

Infection with all chlamydial species promotes secretion of proinflammatory cytokines in response to outer membrane proteins, heat shock proteins, and the chlamydial LPS, all of which engender prominent inflammatory responses (Rasmussen et al., 1997). Indeed, LPS alone may account for several aspects of relevant neuropathology in late-onset dementia of the Alzheimer’s type. For example, *E. coli* LPS injected at low dose into the brains of rats elicits an inflammatory response characterized by increased production of cytokines as well as activation of microglial cells (Hauss-Wegrzyniak et al., 1998); this inflammation is comparable to that currently attributed to A*β* deposition in the late-onset brain (Lue et al., 1996). Interestingly, trials investigating the effects of NSAID treatment implicate inflammation as a pathologic factor in the disease, although they did not demonstrate NSAID treatment to be an effective therapeutic approach following disease onset (Breitner, 1996). Importantly, for a number of reasons it was long thought that chlamydiae did not produce peptididoglycan, although genome analyses have demonstrated genes encoding it, and recent studies from several groups have shown that it is, in fact, produced (e.g., Liechti et al., 2014). Clearly this molecule can and probably does act to induce inflammation in the nervous system and elsewhere.

In the late-onset brain, both activated astrocytes and microglia have been identified around amyloid plaques (Wood, 1998). In our studies, we found infected microglia, astroglia, perivascular macrophages and neurons in areas of amyloid deposition. Identification of *C. pneumoniae* infection in microglia and astroglia in late-onset disease suggests that inflammation initiated by infection might be involved in neuropathogenesis (Balin et al., 1998; Gérard et al., 2006). Both of these cell types respond to insult by producing proinflammatory cytokines and reactive oxygen species. Further activation of microglia and astroglia in response to infected, activated monocytes entering the brain also likely would result in increased production of a variety of cytokines and chemokines (Simpson et al., 1998; Hu and Van Eldik, 1999). Proinflammatory molecules also have been shown to be significantly elevated in supernatant fluids of murine microglial cells infected with *C. pneumoniae* compared with controls, and infected murine astrocytes also showed elevated levels of cytokines, in particular MCP-1 and IL-6, when compared to controls (Boelen et al., 2007b). Conditioned supernatants from infected murine microglial cells increased neuronal cell death when compared to mock-infected supernatants; upon addition of neutralizing antibodies to IL-6 and TNFα to the conditioned supernatant, neuronal cell death was reduced by ~50% (Boelen et al., 2007b).

Observations from our cell culture studies indicate that although transcription of genes encoding inflammatory mediators in *C. pneumoniae*-infected monocytes changes by 48 h post-infection, infected cells maintain pro-inflammatory cytokine secretion of IL-1β, IL-6 and IL-8 over 5 days (Lim et al., 2014). Others have found similar proinflammatory cytokines secreted by monocytes in late-onset disease (Fiala et al., 2005, 2007; Feng et al., 2011; Lim et al., 2014; Saresella et al., 2014). High levels of IL-1β are correlated with neuroinflammation in the late-onset brain (Griffin et al., 1994; Sheng et al., 1996; Serou et al., 1999; Mrak and Griffin, 2001). This cytokine activates nitric oxide synthase, which has been implicated in hippocampal neuronal cell death (Cacabelos et al., 1991; Blum-Degen et al., 1995). Additional evidence has implicated IL-1 cytokines in promotion of the neuronal synthesis of the β-amyloid precursor protein (Griffin et al., 1994). These observations provide a rationale for triggering events by which Aβ production would be a result of neuropathogenesis, rather than an initializing event.

## Transcription Studies

Our ongoing studies are investigating infection of human astrocytes with *C. pneumoniae* strain AR39 and its affects on the expression of numerous genes related to APP processing, including *ADAM10*, *BACE1*, *PSEN1* and the associated subunits of the γ-secretase complex. Transcriptional changes for these genes have been observed in late-onset brains in conjunction with inflammation (reviewed in Agostinho et al., 2015). In this regard, we suggest that *C. pneumoniae* infection could serve as a pro-inflammatory stimulus to initiate and promote relevant amyloid neuropathogenesis. Interestingly, previous studies demonstrated an interrelationship of *C. pneumoniae* infection and the altered expression of genes for lipid-homeostasis (*APOE*, *ABCA1*, *LPL*, *LRP1*), as well as for cytoskeletal organization (*MAPT*; Alvesalo et al., 2008; Di Pietro et al., 2013b; El Yazouli et al., 2017). These gene changes assure that the pathogen receives sufficient host-derived lipids for the energy-demanding processes of growth and replication. In the process of commandeering the expression of these and other related genes, including those for APP processing, *C. pneumoniae* infection promotes pathogenesis through amyloid generating pathways (Little et al., 2004, 2014). Because astrocytes are known to propagate a pro-inflammatory state in the late-onset brain (Zhao et al., 2011), we suspect that the transcriptional changes observed following infection with *C. pneumoniae* will support the role of infection in promoting a pro-inflammatory response.

### Related Studies

We have begun to investigate whether a direct link exists between this respiratory pathogen and the chronic, pathologic neurodegenerative pathology characteristic of late-onset disease. Utilizing an *in vitro* glial model of *C. pneumoniae* infection, we are asking whether a specific mechanism for the production of relevant neuropathogenesis is identifiable. Additional studies will be required to address whether* in vitro* results can be replicated in an *in vivo* setting.

The seminal studies from our group investigating *C. pneumoniae*’s presence in the late-onset brain demonstrated the involvement of microglial and neuronal cells in addition to astrocytes, indicating that the coordinated response of each cell type may contribute cooperatively to the development of pathology. This coordinated response is especially important to define within the framework of neuroinflammation, since microglial cells and neurons may interact differently through recognition and release of pro-inflammatory molecules based on their heterogenous expression of Nod-like receptors, scavenger receptors, toll-like receptors and complement receptors (Shastri et al., 2013).

Downstream of neuroinflammation, the interdependance of microglia, neurons and astrocytes in producing neuropathology may also manifest from the paracrine signaling of post-cleavage APP byproducts. For example, in recently published studies from others, sAPPα released post-ADAM10 ectodomain shedding of APP was shown to serve neuroprotective roles in other cell types by inhibiting *tau* phosphorylation by GSK3β (Deng et al., 2015) and BACE1 activation (Peters-Libeu et al., 2015). Such diverse interactions between cell types may complicate an isolated glial cell model of *C. pneumoniae*-induced neuropathology, but this elucidation is essential for full understanding of the pathophysiology of *C. pneumoniae* infection in the CNS.

### Expression of Other Relevant Gene Transcripts Resulting From *C. pneumoniae* Infection

In our study of monocyte infection, a number of genes encoding host defense products against bacteria were significantly up-regulated 48 h after *C. pneumoniae* infection (Lim et al., 2014). One of these genes, *DEFB4*, encodes a defensin protein with anti-microbial activity linking the innate and adaptive immune responses (Hollox et al., 2003). A second transcript, from *DMBT1*, is typically up-regulated in response to bacterial activation of NOD2, the intracellular pattern recognition molecule, which activates the NFκB transcription factor (Rosenstiel et al., 2007). The gene product of *DMBT1* acts to hinder bacterial invasion and the LPS—induced activation of the toll-like receptor 4 on the surface of cells.

The transcript encoding MCP1/CCL2, a key chemokine for recruiting monocytes and macrophages (Ubogu et al., 2006), was increased up to 1,000-fold following infection of monocytes with *C. pneumoniae* (Lim et al., 2014). This gene product appears to be an important contributor to the neuroinflammatory process observed in the late-onset brain and is increased in both cerebrospinal fluid and plasma from patients with mild cognitive impairment and dementia compared with controls (Fiala et al., 1998; Galimberti et al., 2006). CCL2 may alter the blood brain barrier to allow increased monocyte migration into brain tissues, as well as affecting production and clearance of Aβ from the brain, and possibly allowing Aβ found in the blood access to the brain (Fiala et al., 1998; Yamamoto et al., 2005; Galimberti et al., 2006). Thus, our studies demonstrating increased CCL2 production during *C. pneumoniae* infection of monocytes has implications for late-onset disease since *C. pneumoniae* has been found in cells resident in the brain as well as in perivascular monocytes and macrophages found around the blood vessels in late-onset disease (Balin et al., 1998; MacIntyre et al., 2003; Gérard et al., 2006; Hammond et al., 2010).

Another set of gene product comprise the inflammasome complex that is associated with toll-like receptors and which mediate the response to both extracellular and intracellular pathogens (Schroder and Tschopp, 2010). We determined that the *NLRC4* inflammasome transcript was significantly up-regulated following infection. Others have shown previously that activation of this particular inflammasome is responsible for activating caspase-1 and IL-1β secretion in response to bacterial infection (Franchi et al., 2009; Pereira et al., 2011). Intriguingly, this same inflammasome complex can be activated by type III secretion systems characteristic of *C. pneumoniae* and other gram-negative bacteria (Miao and Warren, 2010). This secretion system acts to transfer effector proteins from bacteria into the cytosol of the host cell, resulting in the generation of reactive oxygen species. These latter are thought to be involved in assemblage of another inflammasome complex, NLRP3 (Abdul-Sater et al., 2009a,b), which coincidentally has also been shown to be activated by chlamydial infections (Abdul-Sater et al., 2010; He et al., 2010). Further, we observed up-regulation of the *AIM2* inflammasome transcript, which would result in activation of an additional inflammasome complex as a result of detecting double-stranded DNA from the bacteria in the cytoplasm (Fernandes-Alnemri et al., 2009; Hornung et al., 2009).

## Antibiotic Treatment

As *C. pneumoniae* may be involved in induction of late-onset dementia, antimicrobial treatment might constitute a therapeutic approach to eliminate the organism from the brain. One clinical trial used a combination approach with doxycycline and rifampin for such treatment and evaluated the change in the Standardized Alzheimer’s Disease Assessment Scale cognitive subscale (SADAScog) at 6 months as the primary outcome (Loeb et al., 2004); changes in that score at 12 months was a secondary outcome measure. Overall, results indicated less decline in SADAScog score at 6 months in the antibiotic group compared with those receiving placebo, although the 12-month score for both groups was not significantly different. Importantly, less dysfunctional behavior was observed in the antibiotic group at 3 months, and for that group reduced decline in mini-mental status scores was observed at 12 months. No correlations were made to changes in *C. pneumoniae* infection as determined by serum antibody titer analysis and following PCR of blood samples. Interestingly, doxycycline has been found to be correlated with the lowering of neuroinflammation in APP/PS1 transgenic mice, suggesting that it may be acting as an anti-inflammatory agent as well as having antibiotic effects (Balducci et al., 2018). This anti-inflammatory activity well could be responsible for the attenuated decline in mini-mental status scores observed at 12 months in the Loeb et al. (2004) trial. However, similar to antibiotic trials assessing efficacy in obviating atherogenesis and cardiovascular disease in which *C. pneumoniae* has been implicated (Campbell and Rosenfeld, 2014), no meaningful efficacy in amelioration of relevant pathogenesis was demonstrated as an outcome of the late-onset-related antibiotic trial. These failures clearly suggest that an antibiotic treatment regimen for complex disease entities once manifested is not a viable strategy. As with NSAIDS following onset of late-onset dementia (Breitner, 1996), use of antibiotics following disease diagnosis probably is too late to provide meaningful efficacy. Individuals demonstrating evidence of *C. pneumoniae* infection prior to disease onset, or at the mild cognitive impairment stage, may respond differently, and perhaps better, to antibiotic therapy. However, this approach has yet to be tried in a controlled clinical trial setting.

Approaches other than, or in addition to, antibiotic therapy may be helpful in treating late-onset disease. *C. pneumoniae* is well known to persist in various contexts, and a persistent form is implicated in chronic diseases. One possible approach to therapy is to manipulate an immune response that can eliminate intracellular infections. To address this issue, we used a synthetic peptide, acALY18 derived from an 18-mer sequence of the transient receptor channel protein 1 (TRPC1; Thacker et al., 2009). This peptide activates, in part, the NLRP3 inflammasome to combat* C. pneumoniae* infections of monocytes *in vitro* (Thacker et al., 2012). Using a low dose of acALY18, only 12% of the cells remained infected at 24 h post-treatment, compared to 90% of cells left untreated (Thacker et al., 2012). At 48 h post-infection, analysis of the infected cells revealed that 26 innate and adaptive immune gene transcripts were up regulated in the treated cells compared to infected/untreated cells. These transcripts occurred in four functional groups: (1) cytokines, chemokines, receptors and signaling molecules; (2) host defense; (3) anti-bacterial response; and (4) modulators of the tissue response to inflammation. These specific up-regulations appear to be effective in clearing infection from a large percentage of *C. pneumoniae*-infected monocytes. Future studies will address more specific transcript up-regulation and specific protein expression leading to eradication of *C. pneumoniae* infection.

## Animal Models for *C. Pneumoniae* Infection of the CNS

The typical animal models of late-onset disease have utilized humanized transgenic mice that over-express mutants of presenilin and the Aβ precursor protein (Guénette and Tanzi, 1999). In these models, the over-expression of amyloid leads to the development of amyloid plaques in the brain, thus paralleling the pathology typically observed in familial Alzheimer’s disease. Interestingly, recent studies have demonstrated that infecting two different models of humanized transgenic animals separately with different organisms (*Bordetella pertussis*, McManus et al., 2014) and (*Helicobacter pylori*, Wang et al., 2014) both result in increased Aβ-amyloid, although through dissimilar mechanisms. What these models do not address, however, are the initiating or triggering events of late-onset disease wherein mutations of the A*β* precursor protein and presenilin proteins are not evident.

We developed non-transgenic animal models to study the means by which infection might impact the pathogenesis of late-onset disease, independent of predisposing genetic factors (Little et al., 2004, 2005). We utilized the *C. pneumoniae* isolate from a late-onset brain (see above) to infect naïve BALB/c mice to assess if infection would promote brain damage similar to that identified in human disease. BALB/c mice previously had been found to be susceptible to respiratory infection with *C. pneumoniae* (strain AR-39), which would produce a persistent infection (Laitinen et al., 1996). Thus, we tested the hypothesis that *C. pneumoniae* infection by a natural route of inoculation in BALB/c mice might trigger processes resulting in development of relevant brain neuropathology (Little et al., 2004). Following intranasal infection, identification of *C. pneumoniae* in the olfactory neuroepithelia (chlamydial antigens) and the olfactory bulbs (chlamydial antigens and chlamydial bodies) was confirmed by both light and electron microscopy (Little et al., 2004). Analysis of the brain revealed pathological Aβ_1–42_ deposits that reflected amyloid plaques seen in human disease. Activated astrocytes in addition to reactive astrocytes co-localized with amyloid deposits, suggesting that a cellular inflammatory response had been initiated. This response may be due to *C. pneumoniae* and/or be directed against amyloid deposits or soluble amyloid induced by *C. pneumoniae* infection. These observations suggest that the infectious insult results in Aβ generation and lend support to the hypothesis that Aβ can act as a “bioflocculant” (Robinson and Bishop, 2002). Induction of amyloid deposits in the non-transgenic BALB/c mouse brain supports the hypothesis that *C. pneumoniae* infection can accelerate or induce relevant neuropathology, and that it can trigger late-onset disease pathogenesis without the early-onset genetic mutations.

Studies evaluating treatment following intranasal infection used antibiotics to determine if this approach could limit the pathology induced by infection in the CNS (Hammond et al., 2006). Following intranasal infection with *C. pneumoniae* (strain AR-39), mice were treated with Avelox (moxifloxacin hydrochloride) for 7–21, 28–42, 56–70, or 84–98 days post-infection; sacrifice was at 6 months post-infection, with brains analyzed for *C. pneumoniae*, Aβ_1–42_ deposition (plaques) and astrocyte (GFAP) cellular reactivity. Immunohistochemistry revealed that the organism (or its antigens) was still present at 6 months post-infection in olfactory tissues and in the brain. At 7–21 days post antibiotic treatment, the number of Aβ_1–42_-reactive amyloid plaques were equal to the level seen in uninfected mice. In infected mice given antibiotic treatment delayed until 56 days post-infection, the amyloid plaque number was 8–9-fold higher than baseline, comparable to the plaque load in the brains of infected animals that received *no* antibiotics. These data validate the need for early antibiotic intervention prior to disease manifestation, and they support our contention that antibiotic treatment of late-onset disease may not be effective following disease diagnosis. Further, they suggest that early antibiotic intervention post-infection is effective in limiting the amyloid plaque formation that arises because of infection, even though complete eradication of the organism or antigens arising there from may not be achieved.

### Additional Animal Model Studies

Our latest animal studies employed the AR-39 laboratory strain for infection rather than *C. pneumoniae* isolates from the human brain. Brains were analyzed at 1–4 months post-infection by immunohistochemistry using *Chlamydia*-specific antibodies and antibodies specific for Aβ_1–42_ (Little et al., 2014). As in our previous report using our human brain isolate of *C. pneumoniae*, no substantial amyloid deposits were found at 1 month post-infection, and only limited relevant amyloid pathology was apparent at 2 months post-infection. In contrast to the original study, however, at 4 months post-infection amyloid pathology was diminished; brains resembled those from mock-infected mice, suggesting that pathology actually had decreased during the 2–4 months post-infection. Interestingly, our analysis indicated that peak chlamydial burden preceded peak amyloid deposition by 1 month, suggesting that *C. pneumoniae* infection can serve as a primary stimulus for the production of Aβ-amyloid and subsequent deposition in animal brain tissues. These observations strongly suggest that the human brain isolates elicit a level of neuropathology that is different from the standard AR-39 respiratory strain of *C. pneumoniae* (see below).

Precedents for infection in the exacerbation of relevant amyloid pathology have been reported for other pathogens in other relevant animal models (McManus et al., 2014; Wang et al., 2014). Once the infection is brought under control though, levels of soluble amyloid apparently decrease, resulting in fewer deposits at 3–4 months post-infection (Hawkes et al., 2012). In mice infected with the brain isolate in our earlier study, amyloid deposits were found as early as 2 months post-infection, with the greatest number identified at 3 months post-infection (Little et al., 2004). Relevant neuropathology developed progressively as both the size and number of amyloid deposits increased from 1–3 months post-infection. Animal models that reflect late-onset disease, however, have been hampered by lack of understanding of the initial factors that promote the early deposition of Aβ-amyloid. Models utilizing direct injection of microbial products have shown induction of transient amyloid production and deposition, suggesting that bacterial products can induce this production (Erickson et al., 2012; Krstic et al., 2012). Interestingly, one previous study did not identify substantial neuropathology in the mouse brain following infection with a respiratory isolate/laboratory strain of *C. pneumoniae* (Boelen et al., 2007a). The authors of that report noted that discrepancies could have been the result of use of the laboratory strain of *C. pneumoniae*, suggesting a difference in virulence properties than that of the human brain isolate used in our previous study. Given that our findings with laboratory isolate AR-39 of *C. pneumoniae* also resulted in less pathology than when using a human brain isolate, we concur with the interpretation of differences in virulence properties between these strains (Little et al., 2014).

Our observations further indicate that *C. pneumoniae* infections differ in their ability to establish persistence and promote progressive neuropathology as a function of age and dosing. A critical issue in development of late-onset disease is age, and by extension, the age at which *C. pneumoniae* infection occurs. An earlier study from our group suggested that brain infection in older animals is readily established following exposure to* C. pneumoniae* (Little et al., 2005). In other studies, aged C57BL/6 mice as compared to young counterparts had a greater propensity to develop chronic and/or progressive respiratory infections following intranasal infection with *C. pneumoniae* (Eddens et al., 2012). A heptavalent CTL epitope minigene vaccine conferred equal protection in the lungs of both young and older mice. However, although the vaccine partially protected against infection spread to the cardiovascular system of young animals, it failed to provide protection in aged animals (Eddens et al., 2012). These data suggest that vaccine strategies targeting the *C. pneumoniae*-specific CTL response are protective for respiratory infection in both young and old animals; however, the vaccine used was ineffective in preventing dissemination to the cardiovascular system in aged mice, or in controlling replication of organism in these tissues (Eddens et al., 2012).

In a new study, we will address the issue of whether induction of Alzheimers-like neuropathology is a feature exclusive to infection with *C. pneumoniae*, or whether this pathology can be induced following exposure to other chlamydial species. Non-transgenic BALB/c mice will be inoculated intranasally with *Chlamydia muridarum*, a mouse-adapted respiratory isolate of *Chlamydia trachomatis*. Mouse brain tissues will be examined for *Chlamydia*-specific labeling and amyloid pathology. Based on data collected from the previous mouse model with *C. pneumoniae*, we hypothesize that intranasal infection with *C. muridarum* can also induce relevant pathology in the brains of BALB/c mice. Thus, these experiments will address whether or not this organism will actually enter the brain and promote Alzheimers-like pathology as readily as previously observed following infections with *C. pneumoniae*. Additional considerations will include the sex of the animals in our studies. To date we, like most others, have used only female animals in our work, partly because human females appear to be at higher risk for the development of late-onset dementia compared to males (Barron and Pike, 2012). Female transgenic mice also have been shown to accumulate more amyloid as compared to males, and they have significant spatial memory deficits (Gallagher et al., 2013; Sierksma et al., 2013). Whether *C. pneumoniae* infections differ with regard to pathology generated in the brains of non-transgenic female mice compared to those of males remains to be determined.

## Conclusion

**Figure 1:**
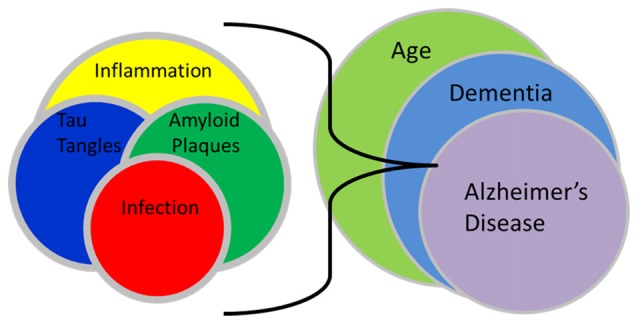
Infection relationship to late-onset dementia. This schematic illustration demonstrates the interactions between infection, amyloid plaques, tau tangles and inflammation with the processes of aging, dementia and Alzheimer’s disease. The interplay of an infection and resultant inflammation can lead to the formation of amyloid plaques and tau tangles which following their accumulation, may promote even more inflammation. These interactions as related to aging may eventually result in a final imbalance that may lead to recognized late-onset dementia of the Alzheimer’s type.

Clearly, much remains to be elucidated regarding the fundamental biochemical, cellular and molecular genetic underpinnings supporting the initiation and development of neuropathology of late-onset dementia of the Alzheimer’s type, and the possible involvement of *C. pneumoniae* in those processes (see Figure 1. Infection relationship to late-onset dementia). We suggest that the difference between progressive and non-progressive neuropathology may be due to uncharacterized differences between/among *C. pneumoniae* strains and host genetic backgrounds. This implies that different virulence factors exist, including those specifying tissue tropism among *C. pneumoniae* isolates and strains. Thus, the ability of the organism to enter and persist in the CNS, and to potentiate a chronic inflammatory response, is crucial to its role in the initiation and maintenance of neuropathogenesis. We emphasize that, as developed in detail in an earlier article, disease definitions should be reconsidered in light of new observations from our group and many other sources. The most obvious neuropathologic aspects, and the disease phenotype, of late-onset dementia are similar to, indeed are functionally identical to, those of early-onset disease. However, all studies to date consistently demonstrate that late-onset disease does not result from lesions in any of the three genes associated with the latter, and extensive research from many groups over the last 30 years has failed to identify any convincing mechanism by which the plaques and tangles are produced specifically in late-onset patients.

Thus, we reiterate here that in our view the age-related dementia referred to as late-onset Alzheimer’s disease should be redefined as late-onset dementia of the Alzheimer’s type. It is functionally unrelated to the familial early-onset, genetically-determined form of dementia properly designated (familial) Alzheimer’s disease. We contend that the etiologies of early-onset dementia and late-onset dementia of the Alzheimer’s type are fundamentally different. Progress in prevention and treatment of increasingly prevalent late-onset disease will be promoted by observing and acting upon this distinction.

Importantly, we suggest that, in concert with other articles in this issue, pathogens in addition to *C. pneumoniae* well may be involved in elicitation of the age-related neuropathology that underlies late-onset disease. As argued in previous publications from this group, complex and largely idiopathic diseases of many types, including rheumatoid arthritis and multiple sclerosis, may be the result of complex and yet poorly understood interactions between various aspects of host genetic background and infectious or other environmental agent(s) (Swanborg et al., 2003; Stanich et al., 2009; Balin et al., 2017). Such complex and multi-faceted interactions will be difficult to elucidate in detail. Indeed, details of those interactions may not be fully congruent among/between the diseases. However, it is abundantly clear at this point that many important diseases under intense current study do not conform to the simplicity of Koch’s postulates, and thus they must be scrutinized with non-traditional modern investigational approaches.

## Author Contributions

All authors contributed to this article, reviewed and approved the final text. BB and AH were the laboratory heads for the initial studies summarized here, but all other authors contributed to the research results to those given in the text as well.

## Conflict of Interest Statement

The authors declare that the research was conducted in the absence of any commercial or financial relationships that could be construed as a potential conflict of interest.
